# Risk of lymph node metastasis in undifferentiated-type mucosal gastric carcinoma

**DOI:** 10.1186/s12957-019-1571-2

**Published:** 2019-02-13

**Authors:** Rieko Nakamura, Tai Omori, Shuhei Mayanagi, Tomoyuki Irino, Norihito Wada, Hirofumi Kawakubo, Kaori Kameyama, Yuko Kitagawa

**Affiliations:** 1 Department of Surgery, School of Medicine, Keio University, 35 Shinanomachi, Shinjuku-ku, Tokyo, 160-8582 Japan; 2 Center for Endoscopy, Kawasaki Municipal Ida Hospital, Kawasaki, Japan; 3 Department of Pathology, School of Medicine, Keio University, Tokyo, Japan

**Keywords:** Undifferentiated adenocarcinoma, Mucosal gastric cancer, Lymph node metastasis

## Abstract

**Background:**

Endoscopic resection (ER) has come to be recognized as a standard treatment for early gastric cancer (EGC). While its adoption is expanding, ER remains restricted to cases of EGC without lymph node metastasis for the treatment of local resection. On the other hand, histopathological analyses of surgically resected specimens of EGC have revealed the presence of lymph node (LN) metastasis in some cases of mucosal gastric cancer (MGC) and undifferentiated MGC (UD-MGC) is considered to have higher risk of nodal metastases than differentiated MGC (D-MGC). To evaluate the risk factors for LN metastasis in MGC, we investigated the characteristics of UD-MGC associated with LN metastasis.

**Methods:**

Among all UD-MGC patients who underwent surgery as initial treatment, between January 2000 and March 2016, we reviewed the clinicopathological data, including the preoperative endoscopic findings and histopathological findings in the resected specimens, of the 11 UD-MGC patients who were identified as having lymph node metastasis. Furthermore, in comparison with cases without lymph node metastasis, we examined the possibility of expansion of the indication for local treatment.

**Results:**

In most of the cases of UD-MGC with LN metastasis, the lesions were relatively large (> 20 mm in diameter) and of the clearly depressed type with faded color and apparent border, and histopathology revealed a high percentage of cases with lymphatic invasion and a predominance of signet ring cell carcinomas. No cases with LN metastasis without depressed macroscopic type nor signet ring cell carcinoma component existed. A degree of invasion of lamina propria (LP) or muscularis mucosae (MM) had same relation to the risk of LN metastasis.

**Conclusions:**

In this study, none of the cases of undifferentiated-type mucosal cancer (UD-MGC) with LN metastasis satisfied the current adoption criteria for ER. We suggested significant risk factors for LN metastasis in UD-MGC cases as depressed tumor type, presence of a signet ring cell carcinoma component, presence of lymphatic tumor invasion, and a large tumor size. More detailed analyses of the endoscopic and histopathological findings may allow further risk classification for LN metastasis in cases of UD-MGC.

## Background

Endoscopic resection (ER) has come to be adopted as a standard treatment method for cases of early gastric cancer (EGC) without lymph node (LN) metastasis, in place of conventional radical gastrectomy with LN dissection. Adoption of ER is based on the criteria described in the Gastric Cancer Treatment Guideline (after 4th edition), and there has been a tendency for expansion of the criteria for adoption of ER for lesions with a low risk of LN metastasis [1, 2]. But still, when postoperative histopathological diagnosis is proven to meet to be within absolute criteria and expanded criteria, the cases are diagnosed not to need additional treatment.

Mucosal gastric cancer (MGC) is also known to be associated with a small risk of LN metastasis, depending on the histopathological pattern, presence/absence of lymphatic/vascular invasion, size, and presence/absence of ulceration. The reported rates of LN metastasis in cases of MGC range from 0 to 21% [3–5]. Current criteria for adoption of ER, including the expanded criteria, are based on the analysis of the groups of patients to have an absent or lower risk of LN metastasis rather than on the risk of mortality from surgery [6], though indications for ESD in undifferentiated adenocarcinoma are still controversial regarding to the oncologic features of undifferentiated EGC.

Surgical gastrectomy is considered the old-standard treatment for patients with undifferentiated-type EGC (UEGC) [7, 8]. However, previous surgical studies showed that the presence of lymph node metastasis was negligible in patients with UEGC, provided the lesion was within expanded indications on final resection pathology [1, 9].

In this study, we evaluated the characteristics of UD-MGC associated with LN metastasis, explored risk stratification for LN metastasis in patients with MGC, and considered whether expansion of adoption for ESD is possible or not on UD-MGC including evaluation of risk of LN metastasis on UD-MGC. Reconsideration of characteristics of UD-MGC with LN metastasis stated in previous study was conducted, and the type of the lesion by endoscopic findings was added to contents of evaluation compared to previously reported study.

## Methods

This retrospective study was conducted to analyze the characteristics of UD-MGC with LN metastasis and to classify risk of LN metastasis by endoscopic findings. Among all the MGC patients who underwent gastrectomy with LN dissection between January 2000 and March 2016 at the Department of Surgery of the School of Medicine at Keio University, a total of 11 patients who had pure or predominantly undifferentiated-type MGC (UD-MGC) were identified as having LN metastasis by histopathological examination of the resected specimens. Meanwhile, among all the MGC patients who underwent gastrectomy with lymph node dissection during the said period, 326 were diagnosed as having UD-MGC. None of these patients with UD-MGC was identified as having LN metastasis preoperatively based on the findings of imaging studies such as CT. However, the results of histopathological examination of the resected specimens revealed that 3.3% of the UD-MGC patients had LN metastasis. In this study, we analyzed the endoscopic and clinicopathological characteristics of these cases of UD-MGC with LN metastasis, and in comparison with cases without lymph node metastasis, we examined the possibility of expansion of the indication for local treatment.

We evaluated the preoperative endoscopic findings (macroscopic type and characteristics of the lesion), histopathological features of the resected specimens, tumor size, extent of tumor invasion, prevalences of lymphatic and vascular invasion, and number of metastatic LNs in the UD-MGC patients diagnosed as having LN metastasis and compared the findings with those in the UD-MGC patients without LN metastasis operated upon during the same period as initial treatment.

The subjects of this study were limited to patients who underwent radical gastrectomy during the above-described period, including those who underwent additional resection after ER, in whom the final pathological diagnosis was MGC.

To evaluate the risk factors for LN metastasis in cases of UD-MGC, we compared the macroscopic lesion type, dominant histopathological pattern, tumor size, prevalences of lymphatic and vascular invasion, and number of retrieved metastatic LNs between the UD-MGC patients with and without LN metastasis. Statistical comparison between the two groups was conducted using the Mann-Whitney *U* test for categorical variables.

In addition to the above variables, we reviewed the endoscopic findings, histopathological pattern, number of metastatic LNs, and depth of tumor invasion (classified as lamina propria (LP) or muscularis mucosae (MM)) in the UD-MGC patients identified as having LN metastasis, and the cases were limited to with lymph node metastasis, whose histopathological appearance was strictly examined to estimate factors of lymph node metastasis.

## Results

### Endoscopic findings of cases of UD-MGC with LN metastasis

**Fig. 1:**
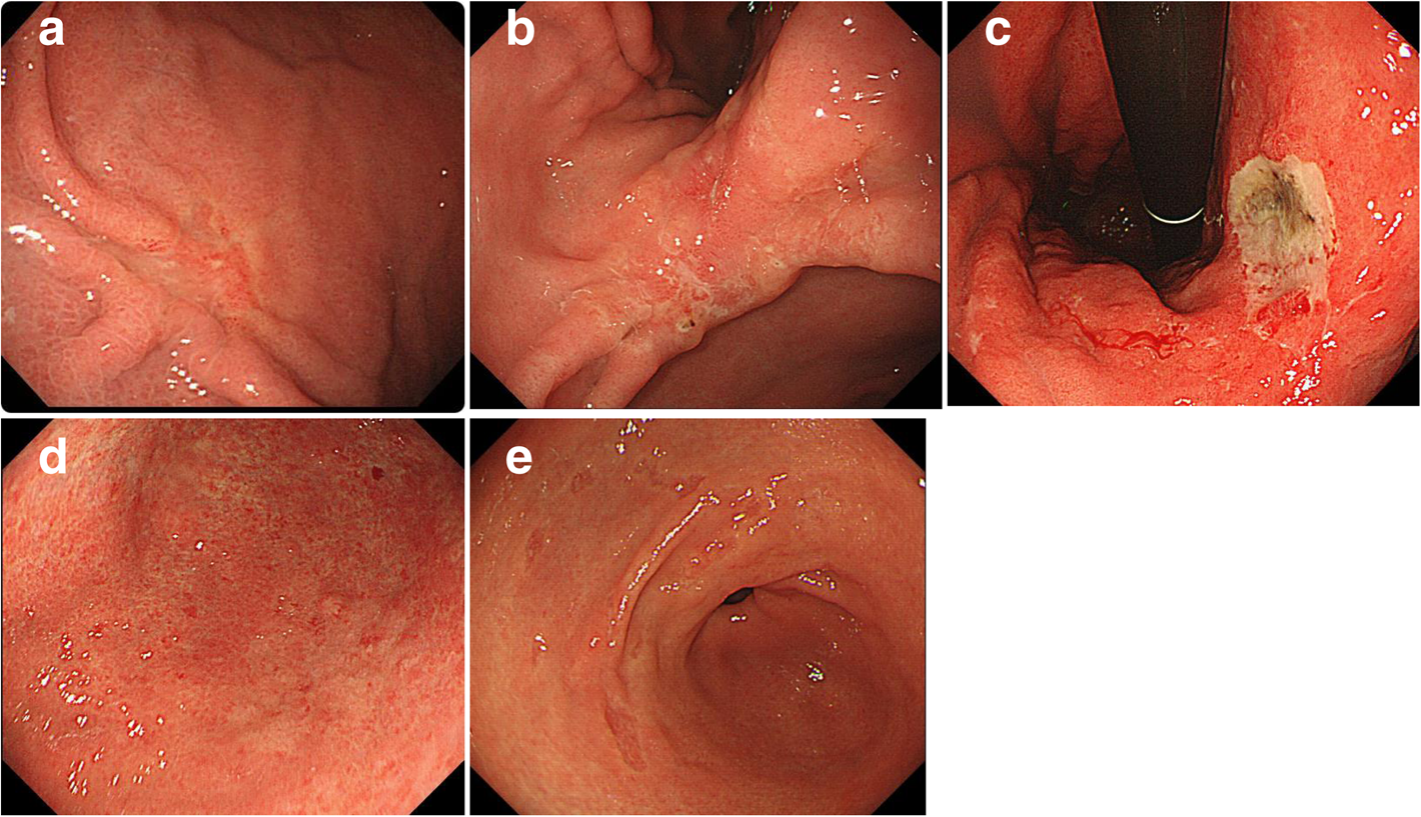
Endoscopic classification with reference to Table 1: **a** type A, **b** type B, **c** type C, **d** type D, and **e** type E

The preoperative endoscopic findings in the cases of UD-MGC with LN metastasis could be classified into the following five types (Fig. 1, Table 1): type A: typical faded, slightly depressed lesions (*n* = 5; most common type); type B: slightly depressed lesion with ulceration (*n* = 2); type C: apparently deep, ulcer-like lesion (*n* = 2); type D: flat, faded lesion (*n* = 1); and type E: unusual lesion whose morphology changed over time and eventually looked like the initial faded, small, depressed lesion (*n* = 1).

**Table 1:** UD-MGC cases with LN metastasis were grouped by the difference of the endoscopic findings to five groups

Type	Findings	Number
A	Typical faded slight depressed lesion	5
B	Slightly depressed lesion with ulcer	2
C	Apparent deep ulcer-like lesion	2
D	Flat faded lesion	1
E	Collected faded small depressed lesion in a circle	1

### Comparison of the characteristics of cases of UD-MGC with and without LN metastasis

Table 2 shows the clinicopathologic characteristics of cases of UD-MGC with and without LN metastasis. The macroscopic tumor type, histopathological pattern, tumor size, and the prevalences of lymphatic and vascular invasion were compared between the two groups. There were significant differences between the two groups in tumor size (*P* = 0.0145) and the prevalences of lymphatic and vascular invasion (*P* < 0.001), while the macroscopic tumor type and histopathological pattern had no significant differences. The results were in accordance with the indication for endoscopic treatment in the gastric cancer guideline that size and lymphatic and vascular invasion are related to endoscopic treatment indication.

**Table 2:** The clinicopathologic characteristics of UD-MGC with LN metastasis and without LN metastasis

	LN(+) (*n* = 11)	LN(−) (*n* = 315)	*P* value
Type			
0-I	0	3	0.699
0-IIa	0	11	
0-IIb	1	5	
0-IIc	10	295	
0-III	0	1	
Histopathological diagnosis			
por1	1	33	0.697
por2	0	53	
por	1	16	
sig + por	3	9	
sig	6	203	
muc	0	1	
Size(mm)			
< 20	0	105	*0.014*
21–30	5	99	
31–40	3	44	
41–50	0	34	
51–60	0	16	
61–70	2	4	
70<	1	0	
Lymphovascular invasion			
ly(−)v(−)	9	311	*< 0.001*
ly(+)v(−)	2	3	
ly(−)v(+)	0	0	
ly(+)v(+)	0	1	

Data in italics are statistically significant

### Detailed analysis of UD-MGC with lymph node metastasis cases

Detailed information about the 11 cases of UD-MGC with LN metastasis is shown in Table 3. Lymphatic invasion was seen in two patients, and the number of retrieved LNs which were metastatic was higher in these two patients than in most of the UD-MGC cases in whom no lymphatic invasion was evident. The lymph node metastasis was classified as N2 or N3 in both cases that showed lymphatic invasion, while only one patient without lymphatic invasion had seven or more metastatic lymph nodes (N2), those the tumor size in this case was large, approximately 70 mm in diameter. In terms of the depth of invasion, invasion up to the lamina propria (LP) was seen in five cases, while invasion up to the muscularis mucosae (MM) was seen in the remaining six cases of UD-MGC with LN metastasis. Histopathologically, nine cases of UD-MGC with LN metastasis had predominant signet ring cell carcinoma, while the remaining two cases had predominantly poorly differentiated adenocarcinoma. No cases with LN metastasis without depressed macroscopic type nor signet ring cell carcinoma component existed.

**Table 3:** Detailed information of 11 patients of UD-MGC with LN metastasis

Case	Age	Sex	Type	Size (mm)	Pathological diagnoses	ly	v	Depth of invasion	Number of LN meta	Number of LN meta	Finding of GIF
1	57	F	IIc	30	sig+por>> tub2	+	−	MM	4, 6, 7, 9	13	A
2	70	M	IIb	35	sig>por	+	−	LP	3a, 4sb, 4d, 5, 6, 7	23	D
3	43	M	IIc	65	por1>sig> tub2	−	−	LP	3, 7	4	B
4	65	F	IIc	70	sig	−	−	MM	3, 5	14	A
5	73	M	IIc	30	sig>>por2	−	−	MM	7	1	C
*6*	63	F	IIc	22	sig+por> tub2	−	−	MM	7	1	A
7	46	M	IIc	40	sig>por2> tub2	−	−	MM	3b	1	B
8	64	M	IIc+IIb	100	por>tub2	−	−	MM	9	1	C
*9*	57	F	IIc	25	sigpor>tub2	−	−	LP	4sb	2	A
*10*	61	M	IIc	30	sig	−	−	LP	6	1	E
*11*	75	F	IIc	25	sig>por	−	−	LP	3a	1	A

### Considering further expansion of the criteria for adoption of ER for UD-MGC: from the viewpoint of tumor diameter and type of tumor

**Fig. 2:**
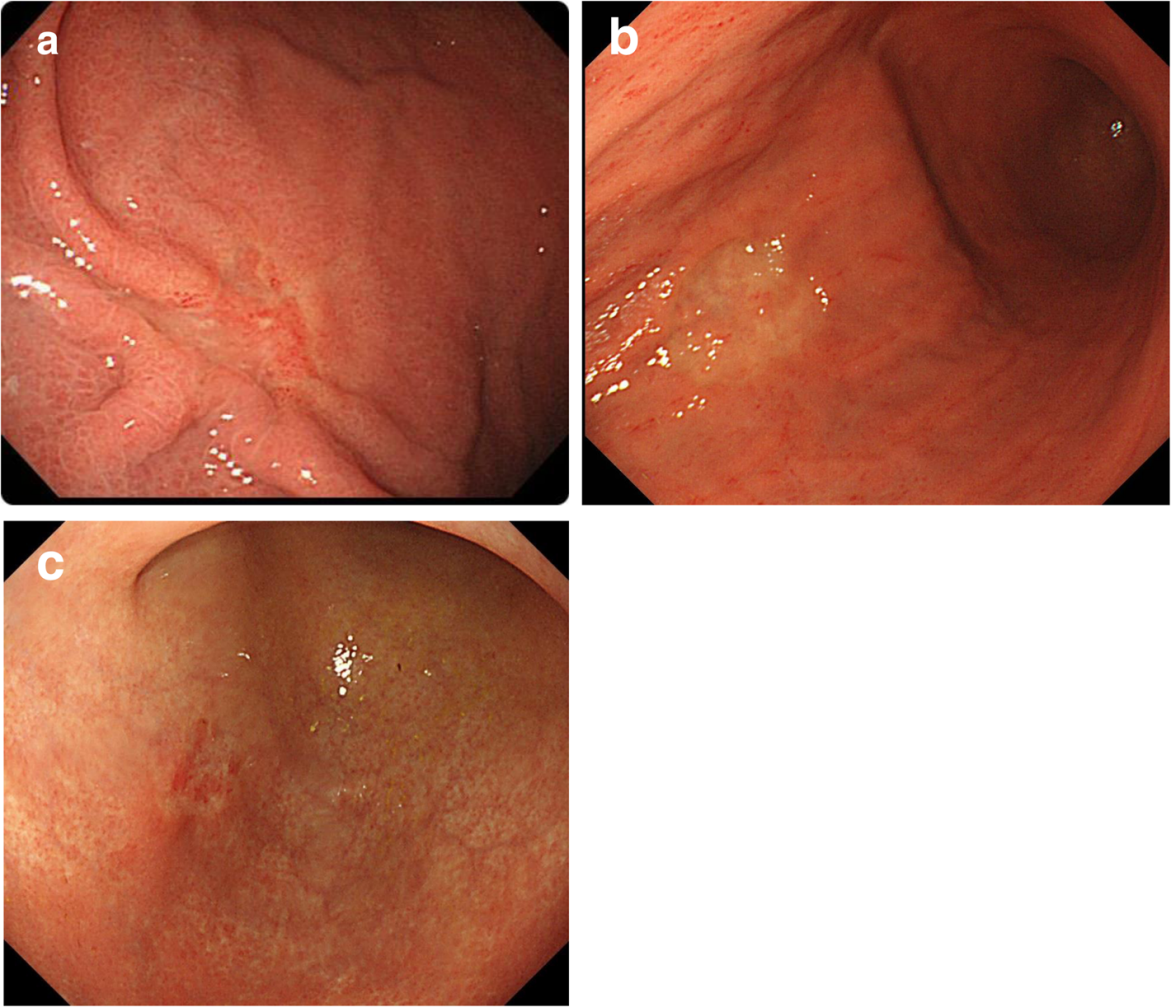
Endoscopic view in patients of UD-MGC with LN metastasis, no ulceration, tumor diameter 21–30 mm, no lymphatic invasion, and no vascular invasion: **a** case 6, **b** case 9, and **c** case 11

**Fig. 3:**
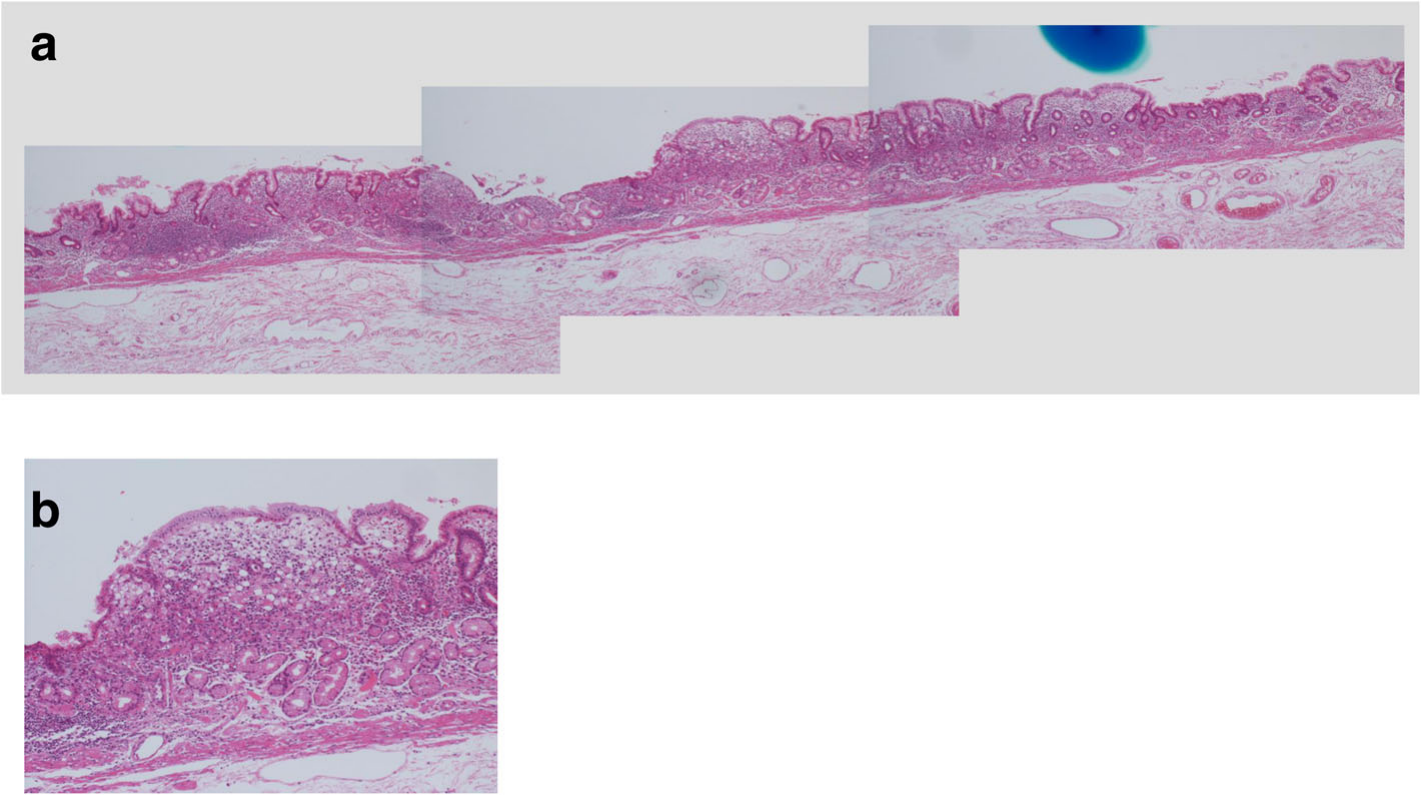
**a**, **b** Histopathological findings of the surgically resected specimen from case 11: the signet ring cell carcinoma is mainly confined to the upper layer of the mucosa

**Fig. 4:**
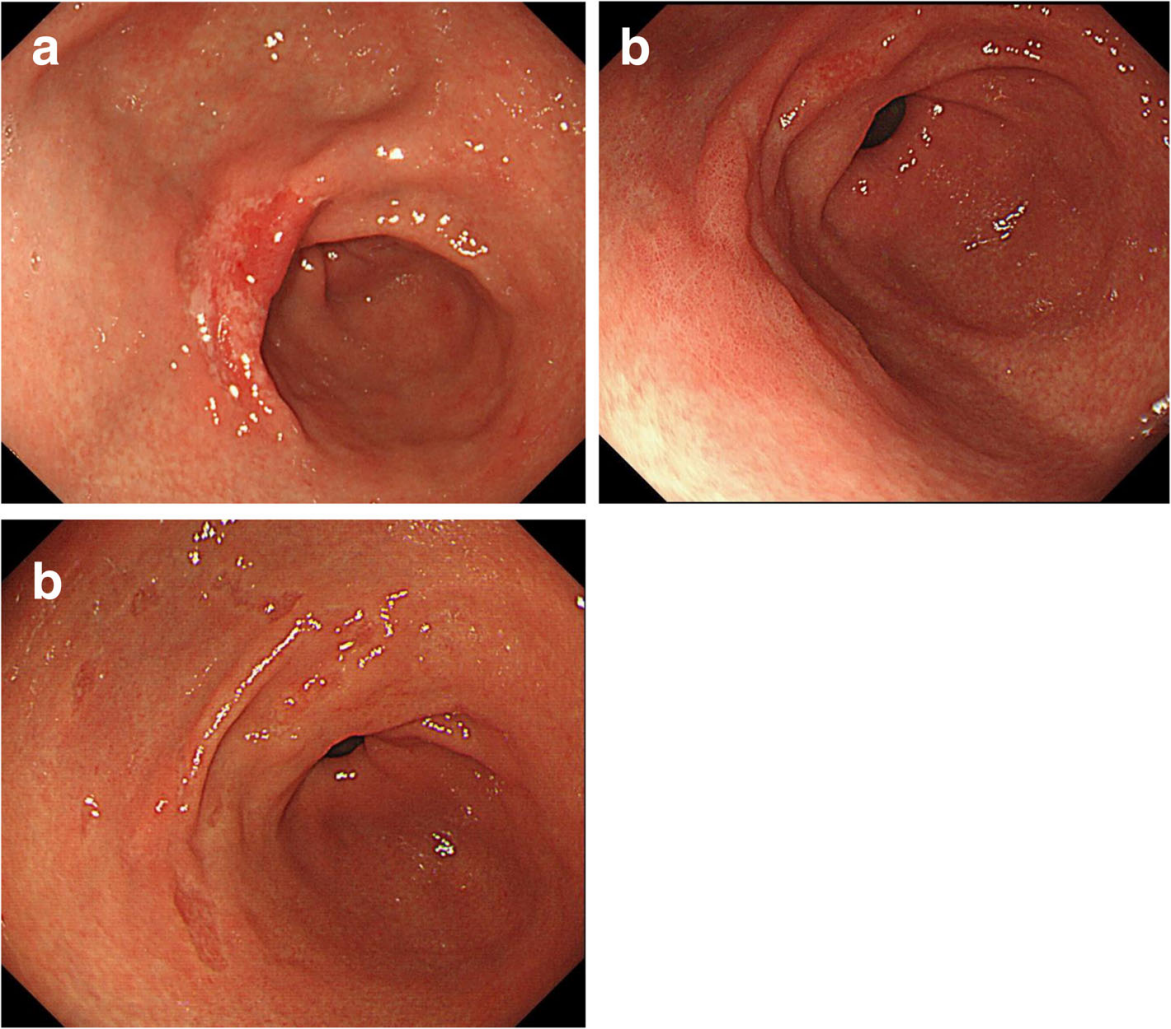
Endoscopic appearances in case 10, which changed with time: at the first endoscopy (**a**), at the endoscopy performed 2 months later (**b**), and at the endoscopy performed 4 months later (**c**)

**Fig. 5:**
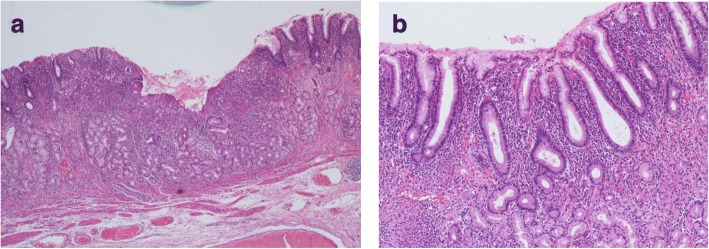
**a**, **b** Histopathological findings of the surgically resected specimen from case 10: the signet ring cell carcinoma exists under normal epithelium

Case nos. 6, 9, 10, and 11 (italicized) with UD-MGC with LN metastasis shown in Table 3 failed to satisfy the criteria for adoption of ER (no ulceration, tumor diameter ≦ 20 mm, no lymphatic invasion, no vascular invasion) [6]. They were excluded only on the basis of the tumor diameters (21–30 mm), which were a little larger than the specified criterion. There is a possibility that ER could be adopted for such cases on the basis of the preoperative findings, diagnosed the size was smaller. Almost all of these four patients showed type A endoscopic findings, tumor type 0-IIc, predominance of signet ring cell carcinoma on histopathology, and only one or two metastatic lymph nodes. Figure 2 shows the findings of the three patients with type A endoscopic findings, namely, slightly depressed lesions with faded color and a clear boundary. Figure 3 shows the histopathological findings of case 11 (Fig. 2c); the signet ring cell carcinoma is mainly confined to the upper layer of the mucosa. There was no evidence of lymphatic invasion or vascular invasion. Figure 4 shows the endoscopic findings of case 10, which were classified as type E, with the endoscopic appearance of the lesion changing between the first endoscopy, the second endoscopy conducted 2 months later, and the third performed 4 months later. Figure 5 shows the histopathological findings of this case; it was identified as a mucosal cancer composed of purely signet ring cell carcinoma, and in some areas, the epithelium overlying the cancer lesion was normal.

### UD-MGC cases with N3 lymph node metastasis: high risk of lymph node metastasis

**Fig. 6:**
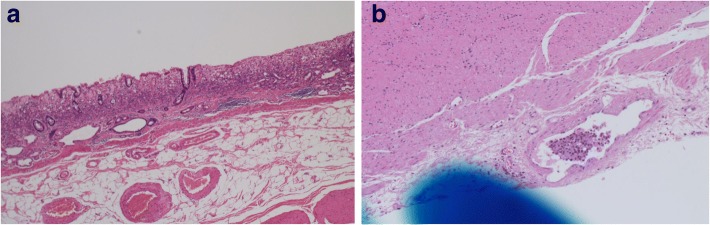
Histopathological findings of the surgically resected specimen from case 2. **a** Signet ring cell carcinoma invading the LP. **b** Lymphatic invasion was observed in the SS layer, but not the SM layer

Only one case (case 2) showed a flat lesion with a faded color (type D) (Fig. 1d); histopathology revealed 23 metastatic LNs in this case. This main lesion in this case was a signet ring cell carcinoma invading the LP (Fig. 6a), although lymphatic invasion was observed in the SS layer, but not the SM layer, of the resected specimen (Fig. 6b). Lymphatic invasion of the SS layer could not have been confirmed if ESD had been performed. The lesion size was 35 mm, which was out of the criterion range for adoption of ER, but it can be cited as an example to underscore the fact that easy adoption of ER for cases of UD-MGC may be dangerous because of the relatively high risk of LN metastasis.

## Discussion

While most cases of MGC do not show LN metastasis, LN metastasis has been confirmed in a small percentage of MGC cases. LN metastasis is the most important factor in cases of MGC for deciding the treatment strategy and determining the prognosis [10–12]. However, in most cases, the LN metastasis is difficult to diagnose preoperatively by CT, etc., and the LN metastasis can only be confirmed by postoperative histopathological examination of the resected specimen. Recently, ER has come to be accepted as a valid minimally invasive treatment for patients with EGC, and the criteria for its adoption are expanding. In cases where the criteria for adoption of ER, including the expanded criteria, are satisfied, ER should be attempted as minimally invasive treatment. In such cases, however, it is important to eliminate the possibility of LN metastasis. Especially, in cases of UD-MGC, even if the criteria for adoption of ER, including the expanded criteria, are fulfilled, the risk of LN metastasis is estimated to be higher than that in cases of D-MGC. Current criteria for adoption of ER are based on a low or absent risk of LN metastasis rather than on the basis of the mortality from surgery [6]. In fact, while many cases who were excluded as candidates for ER were proved by postoperative histopathology after surgical resection to have no residual cancer or LN metastasis, which means additional surgical resection would not have been necessary if ER had been performed in these cases, in some cases, the surgically resected specimens showed residual cancer or LN metastasis. For expanding the current indications for adoption of ER, the characteristics of UD-MGC with LN metastasis should be clarified, and cases with these characteristics should be excluded as candidates for ER. While this was a retrospective study, 11 of 326 patients (3.3%) with UD-MGC had LN metastasis, and all of them had been excluded as candidates for ER. This rate was consistent with the incidence rate of LN metastasis of 2.9 to 9.2% reported previously in cases of UD-MGC [2, 13–17]. According to histopathological analysis, in most cases of UD-MGC with LN metastasis, the histopathological pattern was predominantly signet ring cell carcinoma. Considering that the presence of the signet ring cell carcinoma component has been reported as a poor prognostic factor, predominance of the signet ring cell carcinoma component may be an important predictor of LN metastasis in cases of UD-MGC [15, 16, 18, 19]. Furthermore, cases that showed lymphatic invasion had more metastatic LNs than those without lymphatic invasion. Cases without lymphatic invasion showed only one or two metastatic LNs metastasis, except for one case with a large tumor diameter (70 mm) in which the LN metastasis was classified as N2. According to one systematic review, younger age (< 57 years), tumor located in the middle third of the stomach, large tumor size, depressed tumor type, presence of ulceration, undifferentiated-type histology, and presence of lymphatic invasion are factors significantly associated with the presence LN metastasis in cases of mucosal cancer [20]. We did not evaluate the age or location of the tumor in our study and confined our analysis to cases with undifferentiated-type MGC, although factors such as a large tumor size, depressed tumor type, and presence of lymphatic invasion were also identified as possible risk factors for LN metastasis, consistent with the aforementioned report.

As further histological consideration, the depth of invasion of the tumor has been discussed in some papers. In cases of mucosal esophageal squamous cell carcinoma, ER is indicated only for tumors confined to the LP [21], because of the higher risk of LN metastasis associated with tumors invading the MM. On the other hand, in cases of MGC, it remains unclear whether adoption of ER should be confined to cases of MGC with invasion limited to the LP or can be expanded to include cases with invasion up to the MM. On this point, conflicting reports have been published. Some reports have concluded that MGCs with invasion extending up to the MM show a higher rate of LN metastasis than those with invasion confined to the LP [22, 23], while others have reported the absence of any association between the rate of LN metastasis and the depth of invasion (LP vs. MM) in cases of MGC [1, 24, 25]. Our study revealed a comparable risk of LN metastasis in MGC cases with invasion confined to the LP and those with invasion extending to the MM. However, the number of cases was too small to arrive at any definitive conclusion, and further investigation is needed to evaluate the risk of LN metastasis in relation to the depth of invasion in cases of MGC.

It was difficult to evaluate the depth of invasion in lesions with ulceration, which was an excluded criterion for UD-MGC. In most cases of UD-MGC with LN metastasis, the lesions were of the depressed type with faded color and an apparent border, with a certain degree of invasion of the LP or MM may have the same relation to the risk of LN metastasis. On the other hand, in some cases, undifferentiated adenocarcinoma was sometimes found under normal epithelium, making diagnosis of the lesion difficult. In such cases, if the tumor extent is underestimated or the presence of multiple lesions is judged, ER might be applied. In this study, one case with LN metastasis had such histopathological findings, and one case with a flat lesion without ulceration had N3 LN metastasis, which was suspected to be a result of lymphatic invasion of the SS layer. Caution must be exercised in view of this invasive tendency in UD-MGC cases.

The risk of LN metastasis should not be ignored in cases of UD-MGC, even if the lesions meet the expanded criteria for the adoption of ER. Studies including a larger number of UD-MGC cases, longer-term follow-up, and more detailed analyses of the histopathological findings are needed to confirm the suitability of ER of UD-MGC.

## Conclusion

We identified the following as possible significant risk factors for LN metastasis in cases of UD-MGC in this study: depressed tumor type, presence of a signet ring cell carcinoma component, presence of lymphatic tumor invasion, and a large tumor size. In this study, none of the cases of UD-MGC with LN metastasis satisfied the current adoption criteria for ER. When ER is considered for cases of UD-MGC, the risk of LN metastasis should be borne in mind.
